# Antiproliferative and Apoptosis Induction Potential of the Methanolic Leaf Extract of *Holarrhena floribunda* (G. Don)

**DOI:** 10.1155/2015/756482

**Published:** 2015-03-11

**Authors:** J. A. Badmus, O. E. Ekpo, A. A. Hussein, M. Meyer, D. C. Hiss

**Affiliations:** ^1^Department of Medical Biosciences, University of the Western Cape, New Life Sciences Building, Robert Sobukwe Road, Private Bag Box X17, Bellville, Cape Town 7535, South Africa; ^2^Department of Chemistry, University of the Western Cape, Chemical Sciences Building, Robert Sobukwe Road, Private Bag Box X17, Bellville, Cape Town 7535, South Africa; ^3^Department of Biotechnology, University of the Western Cape, New Life Sciences Building, Robert Sobukwe Road, Private Bag Box X17, Bellville, Cape Town 7535, South Africa

## Abstract

Natural plant products with potent growth inhibition and apoptosis induction properties are extensively being investigated for their cancer chemopreventive potential. *Holarrhena floribunda* (HF) is used in a wide range of traditional medicine practices. The present study investigated the antiproliferative and apoptosis induction potential of methanolic leaf extracts of HF against breast (MCF-7), colorectal (HT-29), and cervical (HeLa) cancer cells relative to normal KMST-6 fibroblasts. The MTT assay in conjunction with the trypan blue dye exclusion and clonogenic assays were used to determine the effects of the extracts on the cells. Caspase activities were assayed with Caspase-Glo 3/7 and Caspase-9 kits. Apoptosis induction was monitored by flow cytometry using the APOPercentage and Annexin V-FITC kits. Reactive oxygen species (ROS) was measured using the fluorogenic molecular probe 5-(and-6)-chloromethyl-2′,7′-dichlorofluorescein diacetate acetyl ester and cell cycle arrest was detected with propidium iodide. Dose-response analyses of the extract showed greater sensitivity in cancer cell lines than in fibroblast controls. Induction of apoptosis, ROS, and cell cycle arrest were time- and dose-dependent for the cancer cell lines studied. These findings provide a basis for further studies on the isolation, characterization, and mechanistic evaluation of the bioactive compounds responsible for the antiproliferative activity of the plant extract.

## 1. Introduction

Cancer remains the leading cause of mortality and morbidity in the world. Annually, about 10 million newly diagnosed cancer cases are reported, of which 6 million result in deaths worldwide [1]. In addition, about 1.6 million new cases and 6 hundred thousand deaths have been predicted for 2013 in the USA alone [2]. Chemotherapy is a major mode of treatment for various cancers, but its success is confounded by unwanted toxic side effects and drug resistance [3–5]. Traditional uses of natural plants to treat different diseases, including cancer, and their scientific isolation and characterization for translation into effective drugs against cancer underscore their importance. This viable source of therapeutic agents from nature might not be unconnected to the structural diversity inherent in a million species of plants and microorganisms but still remains beyond the comprehension of man [6]. Drugs currently in use as anticancer have over 60% of their origin from natural products [7]. Vinblastine (Velban), vincristine (Oncovin), vinorelbine (Navelbine), etoposide (VP-16), teniposide (VM-26), paclitaxel (Taxol), docetaxel (Taxotere), topotecan (Hycamtin), and irinotecan (Camptosar) are some examples of anticancer drugs that had passed through different stages of preclinical and clinical trials and hence were approved for clinical use [8, 9].* Holarrhena floribunda* (G. Don) is a tree that belongs to the Apocynaceae family of plants known to be rich in alkaloids. Alkaloids are compounds found to be responsible for the anticancer activity of the* Camptotheca acuminata* plant, for example, from which the currently used anticancer drug camptothecin is isolated. The leaves of* Holarrhena floribunda* are used in folklore medicine as an antimalarial in Ghana [10]. In Ivory Coast, the bark of this plant is used as a treatment for diarrhoea and the leaves were used as a treatment for amenorrhea [11]. The root is boiled in milk and used to bathe boys attaining puberty and it is also used as a cure for snakebites and venereal disease [12]. Extracts of* Holarrhena floribunda* have been shown to exhibit significant cytotoxic activity compared to other plants screened [13]. Its antioxidant, antimutagenic, and lipid peroxidation inhibition potential have been reported [14]. The present study aimed to evaluate the antiproliferative, apoptosis, and reactive oxygen species inducing activities of the methanolic leaf extract of* Holarrhena floribunda *in breast cancer (MCF-7), colon cancer (HT-29), and cervical cancer (HeLa) cells relative to normal fibroblasts (KMST-6).

## 2. Materials and Methods

### 2.1. Plant Material

Fresh samples of* Holarrhena floribunda* leaves were collected during the raining season in Igbajo, Osun State, Nigeria. Messrs E. C. Chukwuma and O. A. Ugbogu identified the plant at the Federal Research Institute of Nigeria (FRIN). A voucher specimen (FHI 109764) was deposited at the institute herbarium.

### 2.2. Preparation of Methanolic Leaf Extract of* Holarrhena floribunda*

The leaves of* Holarrhena floribunda* were allowed to air-dry at room temperature. The dried leaves were soaked in absolute methanol. The mixture was thoroughly mixed and filtered after 48 h using a Buchner vacuum funnel. The residue was reconstituted in fresh absolute methanol for 24 h and filtered again as described above. The filtered supernatant was evaporated to dryness with a rotary evaporator to eliminate methanol. The weight of pulverized extract of the leaves obtained represents a 21.78% yield in relation to the weight of the leaves used. A stock solution of the extract was prepared by dissolving it first in DMSO followed by reconstitution with growth medium such that the final equivalent concentration of DMSO in the extract was exactly 0.1%.

### 2.3. Maintenance of Cell Culture

Breast cancer cells (MCF-7), colon cancer cells (HT-29), cervical cancer cells (HeLa), and normal human fibroblasts (KMST-6) were obtained from the Department of Biotechnology, University of the Western Cape. The cell lines were maintained in Dulbecco's Modified Eagle's Medium (DMEM) and supplemented with 10% of fetal bovine serum (FBS) and 1% of penicillin and streptomycin (100 U/mL penicillin and 100 *μ*g/mL streptomycin). All tissue culture operations were carried out in a model NU-5510E NuAire DHD autoflow automatic CO_2_ air-jacketed incubator.

### 2.4. Chemicals

3-(4,5-Dimethylthiazol-2-yl)-2,5-diphenyltetrazolium bromide (MTT), trypan blue dye, and crystal violet were purchased from Sigma-Aldrich. APOPercentage dye was obtained from Biocolor Ltd. (Carrickfergus, Northern Ireland), 5-(and-6)-chloromethyl-2′,7′-dichlorofluorescein diacetate acetyl ester (CM-H_2_ DCFDA) from Invitrogen (South Africa), Annexin V-FITC apoptosis kit from BD Pharmingen (USA), and Caspase-Glo 3/7 and caspase-9 assay system kits from Promega (USA). All other chemicals used were of analytical grade.

### 2.5. MTT Assay

The viable cells were seeded at a density of 5 × 10^4^ (100 *μ*L/well) in 96-well plates and incubated in a humidified atmosphere of 5% CO_2_ and 95% air at 37°C for 24 h to form a cell monolayer. After 24 h, the supernatant on the monolayer was aspirated and 100 *μ*L of medium and varying log concentrations of extract (0.1, 1, 10, 100, and 1000 *μ*g/mL) were added and incubated for 24, 48, and 72 h time points. After the specific times of exposure to the extract, 20 *μ*L of 5 mg/mL MTT in PBS was added to each well and incubated for 3 h at 37°C in a 5% CO_2_ atmosphere. Supernatants were removed and 150 *μ*L of isopropanol was added and the plates were gently shaken for 15 min to solubilize the formazan crystals and absorbance was measured at 560 nm using GloMax-Multi Detection System (Promega, USA). The percentage inhibition of proliferation was calculated using the formula below and IC_50_ values were calculated from log dose-response curves using GraphPad Prism software version 6 for Windows (GraphPad Software, La Jolla, California, USA, http://www.graphpad.com/). Consider

(1) %  Inhibition  of  Proliferation=100−Test  Absorbance  at  560 nmUntreated  Control  Absorbance  at  560 nm×100%.

### 2.6. Trypan Blue Dye Exclusion Assay

The viable cells were seeded at a density of 5 × 10^4^ (1 mL/well) in a 12-well plate and incubated in a humidified atmosphere of 5% CO_2_ and 95% air at 37°C for 24 h to form a cell monolayer. After 24 h, culture medium was gently aspirated and treated with 1 mL of medium and varying concentrations of extract (100, 200, 300, 400, and 500 *μ*g/mL) for 24 h. The adherent cells were removed by trypsinization using 0.25% EDTA trypsin (Sigma-Aldrich). The cells were centrifuged using a Bio-Rad tabletop centrifuge at 2,500 rpm for 5 min and the supernatant was discarded to obtain a cell pellet. Cell pellets were resuspended in fresh medium from which a 10 *μ*L aliquot was mixed with an equal volume of 0.4% trypan blue dye and loaded into Bio-Rad TC20 cell counter. Readings were automatically generated from the machine and recorded based on the formula below:

(2) $$\begin{array}{c} \%\;\text{Inhibition}=100-\frac{\text{Total}\;\text{Dead}\;\text{Cell}\;\text{Count}}{\text{Total}\;\text{Cell}\;\text{Count}}\times 100\%. \end{array}$$

### 2.7. Clonogenic Assay

The viable cells were plated at a density of 1 × 10^3^ (1 mL/well) in 6-well plates and allowed to attach for 24 h. After 24 h, the cells were treated with the methanolic leaf extract of* Holarrhena floribunda* (100, 200, 300, 400, and 500 *μ*g/mL) and incubated further for 24 h in a humidified CO_2_ incubator. The medium containing extract was removed and replaced with fresh medium and incubated for another 5 days after which the medium was removed and the cells were washed with PBS. Cells were fixed in 500 *μ*L fixative (75% methanol and 25% acetic acid) for 5 min at room temperature. The fixative was aspirated and the cells were stained with 0.5% crystal violet for 30 min at room temperature. Cells were then washed under running tap water to remove the dye. Colonies were counted and the survival rate was calculated as the percentage of treated over untreated.

### 2.8. APOPercentage Apoptosis Assay

The cells were treated as reported in the trypan blue assay above. Following incubation for 24 h, floating cells in each respective treatment were transferred to 15-mL centrifuge tubes and the adherent cells trypsinized and added to the respective tubes containing floating cells. Cells were washed with 1% PBS and resuspended in residual PBS. APOPecerntage dye (100 *μ*L) in complete culture medium (diluted 1 : 160 v/v) was added to the tubes and allowed to incubate for 30 min at 37°C in a humidified CO_2_ incubator. After the incubation, cells were washed twice by resuspension in 500 *μ*L of 1% PBS and centrifugation for 5 min at 3,000 rpm. The cell pellet so obtained was resuspended in PBS and aliquots were analyzed on a Becton Dickinson FACScan instrument (BD Biosciences Pharmingen, San Diego, CA, USA) fitted with a 488 nm argon laser. A minimum of 10,000 cells per sample were acquired and analyzed using the CellQuest Pro software (BD Biosciences).

### 2.9. Annexin/PI Assay

The cells (2 mL) at a density of 5 × 10^4^ were grown in 40-mm petri dishes and allowed to attach for 24 h after which cells were treated with the 200 *μ*g/mL extract for 12, 24, and 48 h. After the various treatment periods, cells were harvested and centrifuged at 335 g for 10 min. The supernatants were washed in 1% PBS and resuspended in Annexin V binding buffer. The cells were centrifuged at 335 g for 10 min and supernatants were discarded. The cell extracts were suspended in 100 *μ*L Annexin V binding buffer and 5 *μ*L Annexin V Alexa Fluor 488 was added and allowed to incubate in the dark for 15 min. PI (4 *μ*L) diluted in 1x Annexin V binding buffer (1 : 10) was added and allowed to incubate for 15 min in the dark at room temperature. Annexin V binding buffer (500 *μ*L) was added to wash the Annexin/PI stained cells. Annexin/PI was evaluated according to previously described method [15] on a Becton Dickinson FACScan instrument (BD Biosciences Pharmingen, San Diego, CA, USA) fitted with a 488 nm argon laser. A minimum of 10,000 cells per sample were acquired and analyzed using CellQuest Pro software.

### 2.10. Caspases 3/7 and 9

The evaluation of caspase-3/7 was performed according to the manufacturer's instructions. Briefly, 100 *μ*L of 5 × 10^4^ cells/mL was seeded in white-walled 96-well microplates and incubated for 24 h. The cells were treated with the methanolic extract (100, 200, 300, 400, and 500 *μ*g/mL) for 24 h. After treatment, an equal volume of Caspase-Glo 3/7 reagent was added and agitated for 30 sec and the luminescence signal recorded with the GloMax-Multi Detection System (Promega, USA) after incubation for 1 hour. Caspase-9 was evaluated by treating the cells in the same manner as described above.

### 2.11. Cell Cycle Analysis

The cells were seeded at a density of 2 × 10^5^/mL (2 mL/well) in 6-well plates and incubated for 24 h at 37°C in a CO_2_ incubator to form a monolayer. The cells were treated with 200 *μ*g/mL of methanolic extract for 12 and 24 h. After treatment, the cells were washed with 2 mL of PBS and cell pellet was resuspended in 1 mL (1%, w/v) paraformaldehyde in PBS (pH 7.4) on ice for 30 min. The cell pellets were washed twice in 5 mL of PBS. Slowly, 70% of ethanol was added to the cells while vortexing to reduce cell clumping. The cells were stored in −20°C for 48 h after which cells were pelleted at 4000 rpm for 10 min. The cells were then washed in 2X PBS and 1 mL of PI master mix containing 100 *μ*g/mL RNase and 40 *μ*g/mL PI in PBS. Cell cycle phase distribution was determined using a FACSCalibur flow cytometer (BD Biosciences, Franklin Lakes, NJ, USA). The DNA content of 50,000 events was determined by ModFit software (Verity Software House, Topsham, ME), which provided histograms to evaluate cell cycle distribution.

### 2.12. Reactive Oxygen Species

Detection of reactive oxygen species within cells was evaluated using the fluorogenic molecular probe 5-(and-6)-chloromethyl-2′,7′-dichlorofluorescein diacetate acetyl ester (CM-H_2_DCFDA, Invitrogen). Briefly, cells were cultured in 6-well plates at a density of 2 × 10^5^/mL (2 mL/well). The cells were treated with 200 *μ*g/mL of extract for 12 and 24 h. After the treatment, cells were washed with PBS and stained with 7.5 *μ*M of (CM-H_2_DCFDA) prepared in PBS from a DMSO stock solution and incubated for 30 min at 37°C in a humidified CO_2_ incubator. The cells were washed twice with ice-cold PBS after which the cells were acquired and 10,000 events analyzed on a Becton Dickinson FACScan instrument (BD Biosciences Pharmingen, San Diego, CA, USA) fitted with a 488 nm argon laser.

### 2.13. Statistical Analyses

Data are expressed as mean ± SD of experiments performed in triplicate. The values were analyzed by two-way ANOVA followed by Tukey's multiple comparison test using GraphPad Prism software version 6 for Windows (GraphPad Software, La Jolla, California, USA, http://www.graphpad.com/).

## 3. Results

**Figure 1:**
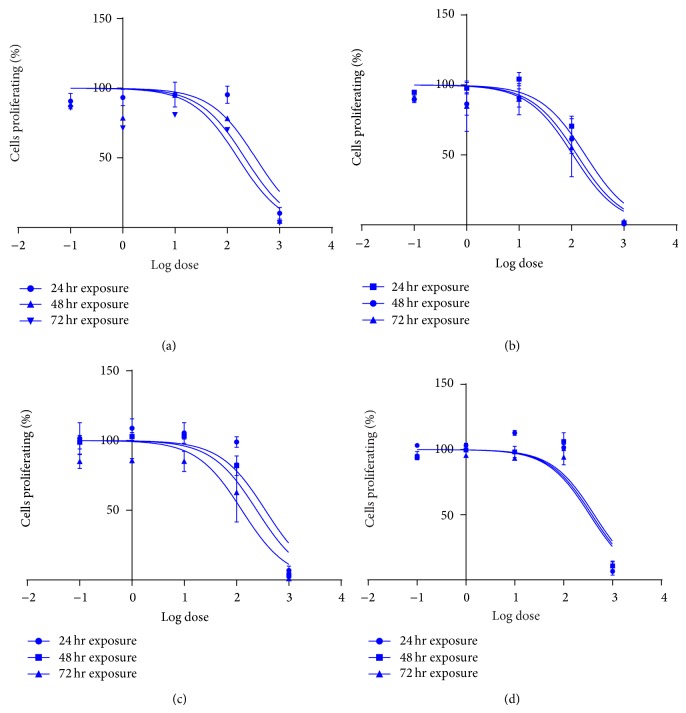
The graphs (a)–(d) show the log dose cytotoxic effects of methanolic leaf extract of* Holarrhena floribunda* in HT-29, HeLa, MCF-7, and KMST-6 cell lines, respectively, for 24, 48, and 72 h treatments. MTT assay was employed to assess the cytotoxic effect of the extract and the graphs were prepared as means ± SD of five separate experiments using GraphPad Prism 6 statistical software.

**Figure 2:**
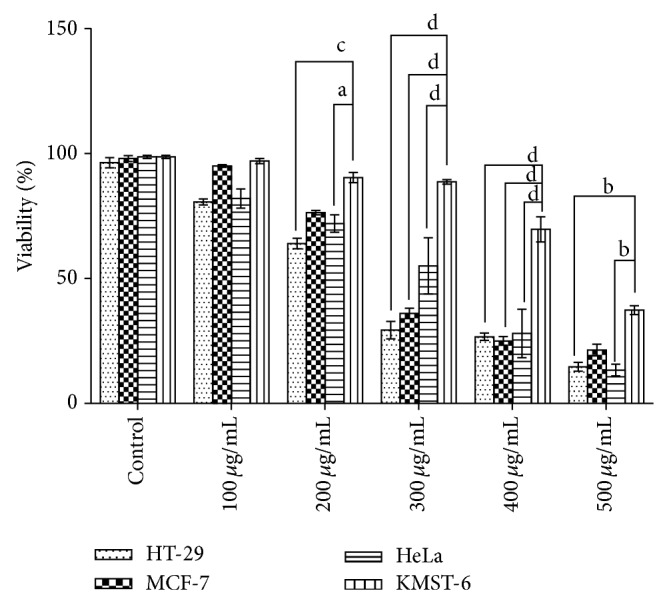
Methanolic leaf extract effect on the viability of cell lines (HT-29, MCF-7, HeLa, and KMST-6) evaluated using the trypan blue exclusion assay. Cell viability was assessed using Bio-Rad TC20 cell counter. The graph represents means ± SD of five independent experiments using GraphPad Prism 6 statistical software while a, b, c, and d represent *P* < 0.5, 0.01, 0.001, and 0.0001, respectively.

The cytotoxic effect of the methanolic extract of* Holarrhena floribunda* was evaluated on HT-29, HeLa, MCF-7, and KMST-6 cells using the MTT assay.  Figure 1 shows the log dose-response curve from which the half-maximal (IC_50_) cytotoxic effects on the extract were estimated by nonlinear regression analysis.  Table 1 depicts IC_50_ values for the 24, 48, and 72 h treatments. The results showed that all the cell lines responded to the cytotoxic effects of the plant extract in a dose- and time-dependent manner. The HeLa cancer cells, however, were more sensitive to the plant extract as shown by its IC_50_ values for 24, 48, and 72 h (182.6, 127.4, and 106.7 *μ*g/mL, resp.). Moreover, the extract exhibited selective cytotoxicity in normal fibroblast cell KMST-6 with higher IC_50_ values of 376.9, 428.2, and 342.3 in 24, 48, and 72 h, respectively. Cell viability was evaluated using the trypan blue dye exclusion assay in cell lines exposed to doses of 100–500 *μ*g/mL of extract. The assay further reaffirms the sensitivity of the cancer cell lines compared to the noncancerous cell (KMST-6) as presented in Figure 2.

**Figure 3:**
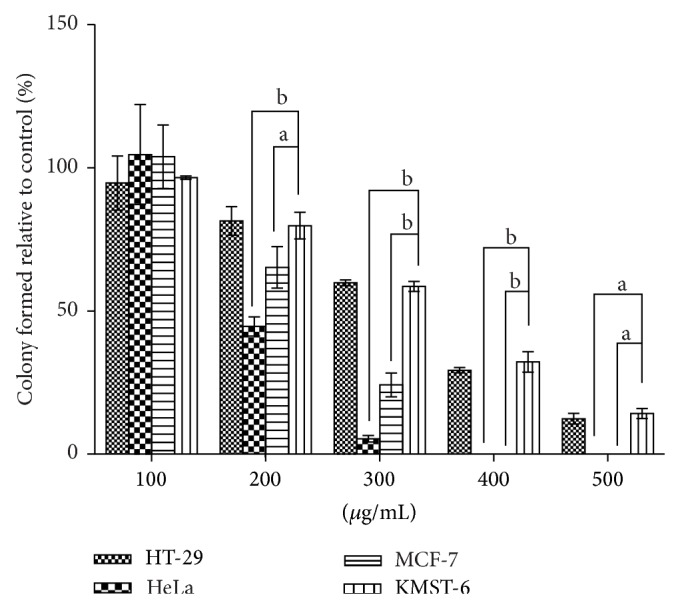
Percentage of colonies formed after the cells were treated for 24 h with methanolic leaf extract of* Holarrhena floribunda*. The paraformaldehyde fixed cells were stained with 0.5% crystal violet dye and the colonies formed were counted using colony counter. Colony formation inhibition at each concentration of the extract is expressed in terms of percentage of control and reported as the means ± SD of five independent experiments while a and b represent *P* < 0.5 and 0.0001, respectively.

**Figure 4:**
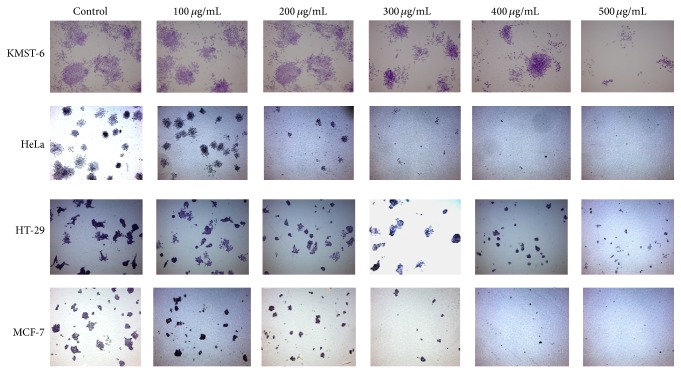
Colony formation ability of the cells following treatment with the extract at different concentrations for 24 h. The pictures were taken using ZEISS Primo Vert microscope and the colonies were counted with colony counter.

The antiproliferative activity of the extract was further evaluated using the clonogenic survival assay. This assay measures the potential of cells to expand into colonies unrestricted by growth contact inhibition—unlike normal growing cells that cease proliferation upon contact inhibition. As presented in Figures 3 and 4, the colony formation declines with increasing concentration of the extract. Remarkably, MCF-7 and HeLa cell lines exhibited no colonies at extract concentrations of 400 and 500 *μ*g/mL.

**Figure 5:**
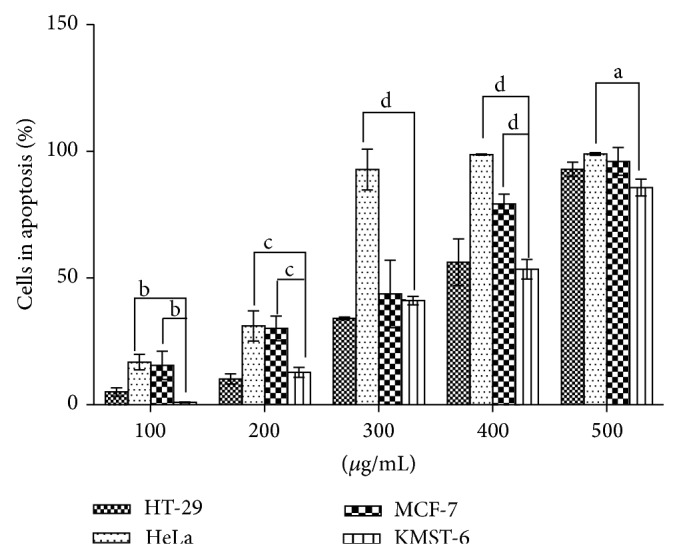
Apoptotic effect of methanolic leaf extract of* Holarrhena floribunda* on cell lines (HT-29, MCF-7, HeLa, and KMST-6). The cells were stained with the APOPercentage dye and evaluated using a flow cytometer. The graph is reported as the means ± SD of five independent experiments using GraphPad Prism 6 statistical software while a, b, c, and d represent *P* < 0.5, 0.01, 0.001, and 0.0001, respectively.

The apoptotic effect of the methanolic extract was explored by staining the cells with APOPercentage dye and evaluation by flow cytometry. The results showed that the induction of cytotoxicity observed occurs through the mechanisms associated with apoptosis. The extract induced apoptosis in a concentration-dependent manner (Figure 5). The HeLa cell line was significantly (*P* < 0.0001) sensitive to the extract when compared with other cell lines. The sensitivity of the KMST-6 cells to the apoptosis-inducing potential of the extract was also low compared to other cell lines. The apoptosis-inducing potential of the extract was further tested in cancer cells using the Annexin-FITC/propidium iodide double staining flow cytometric assay. The cells were exposed to 200 *μ*g/mL of extract for 12, 24, and 48 h.  Table 2 shows that after 24 h of exposure, HT-29 cells had undergone early apoptosis. HeLa cells entered a late apoptotic stage after 48 h while MCF-7 cells exhibited significant necrotic cell at this time period.

**Figure 6:**
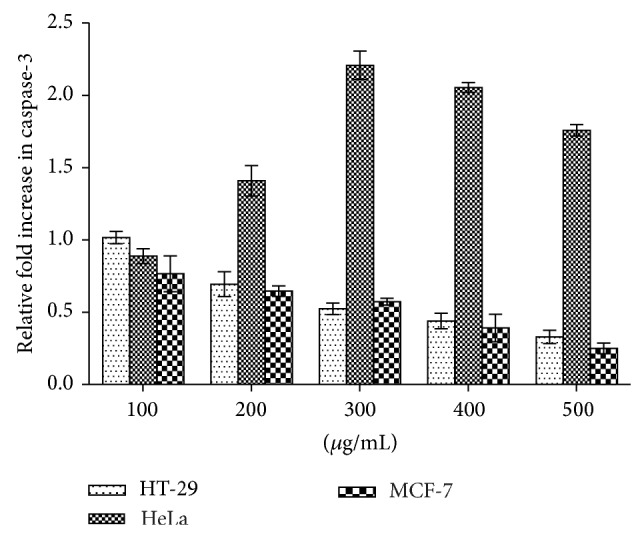
Effects of methanolic leaf extract of* Holarrhena floribunda* on the caspase-3 activation in cell lines (HT-29, HeLa, and MCF-7). Caspase-3 activity was evaluated using Caspase-Glo 3/7 luminescent assay kit (Promega). The graph is reported as the means ± SD of three independent experiments using GraphPad Prism 6 statistical software.

**Figure 7:**
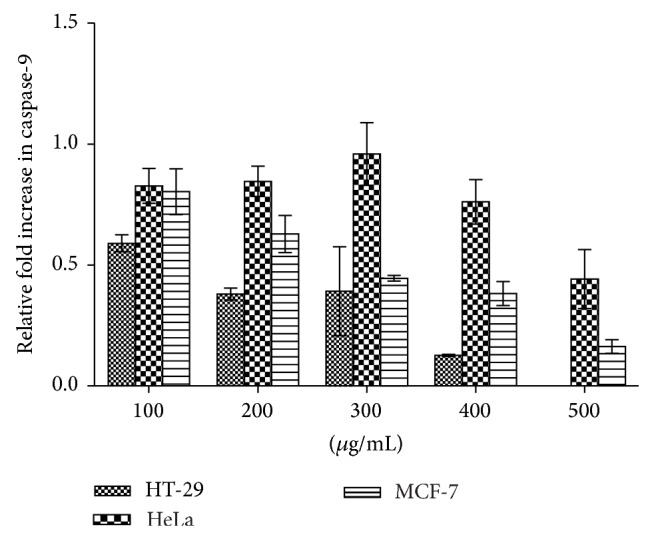
Effects of methanolic leaf extract of* Holarrhena floribunda* on the caspase-9 activation in cell lines (HT-29, HeLa, and MCF-7). Caspase-9 activity was evaluated using Caspase-Glo 9 luminescent assay kit (Promega). The graph is reported as the means ± SD of three independent experiments using GraphPad Prism 6 statistical software.

The activation of caspases 3/7 and 9 was evaluated in cancer cells to establish the cell death pathway induced by the extract.  Figure 6 indicates that caspase-3/7 activity in HeLa cells consistently and significantly increased several folds—in a concentration-dependent manner between 200 and 500 *μ*g/mL of the extract—above those of the MCF-7 and HT-29 cells. Caspase-9 activities in all the cell lines decrease in a concentration-dependent manner (Figure 7).

**Figure 8:**
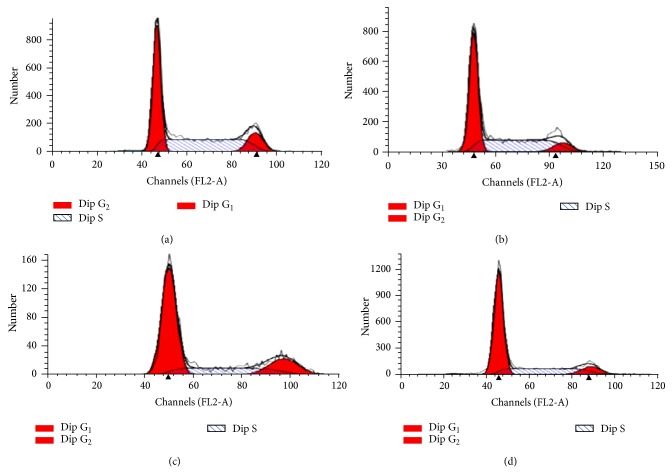
Representative histograms of DNA content distribution of cell cycle phases of HeLa cell treated with methanolic leaf extract of* Holarrhena floribunda* for 12 and 24 h. (a) represents 12 h control cell while (b), (c), and (d) represent 24 h control and 12 h and 24 h cells treated with the 200 *μ*g/mL extract, respectively.

**Figure 9:**
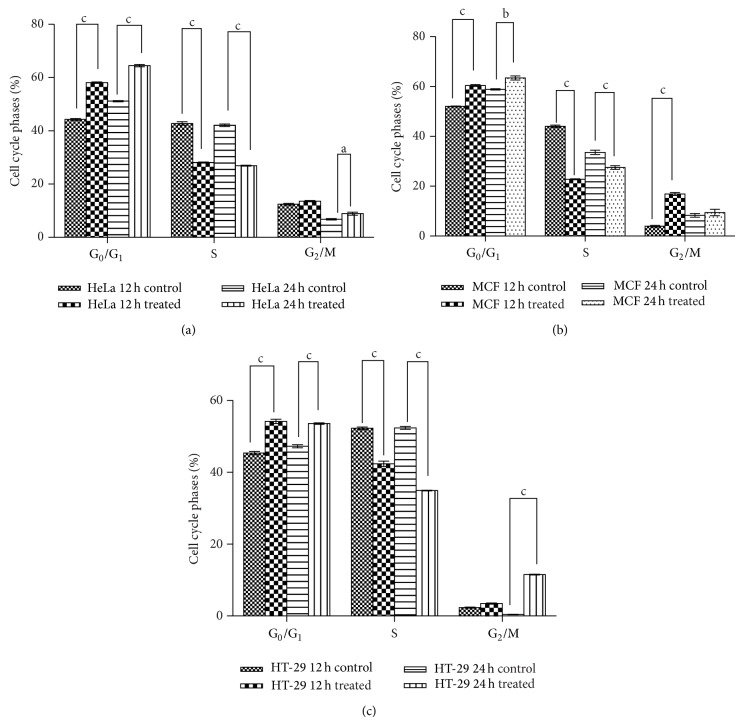
Percentage of HeLa cell (a), MCF-7 (b), and HT-29 (c) in the G_0_/G_1_, S, and G_2_/M phases after incubation with the 200 *μ*g/mL leaf extract of* Holarrhena floribunda* for 12 and 24 h. The values are representative of means ± SD of five separate experiments using GraphPad Prism 6 statistical software while a, b, and c represent *P* < 0.5, 0.01, and 0.001, respectively.

**Figure 10:**
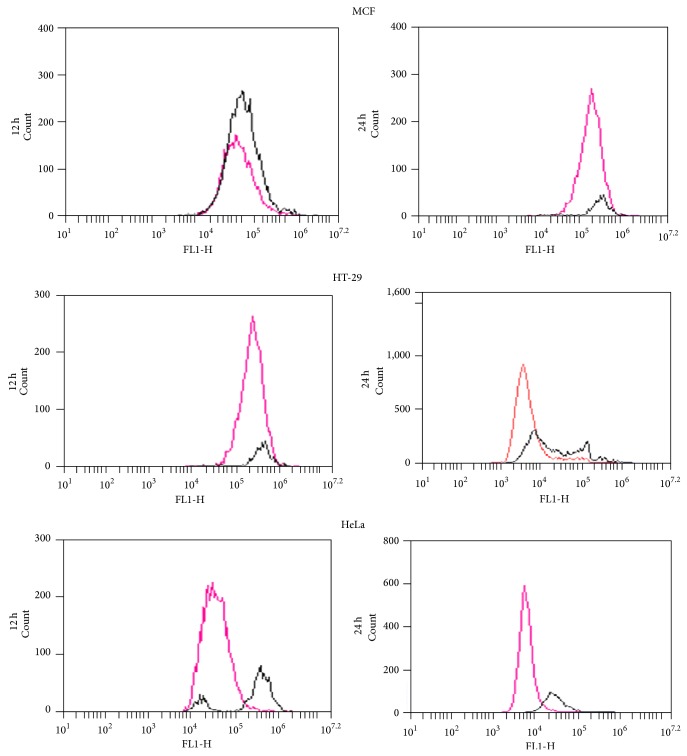
The representative histograms of the cell stained with CM-H_2_DCFDA dye and evaluated using a flow cytometer. The pink histogram depicts stained control cells while black shows the cells treated with the extract at 12 and 24 h.

**Figure 11:**
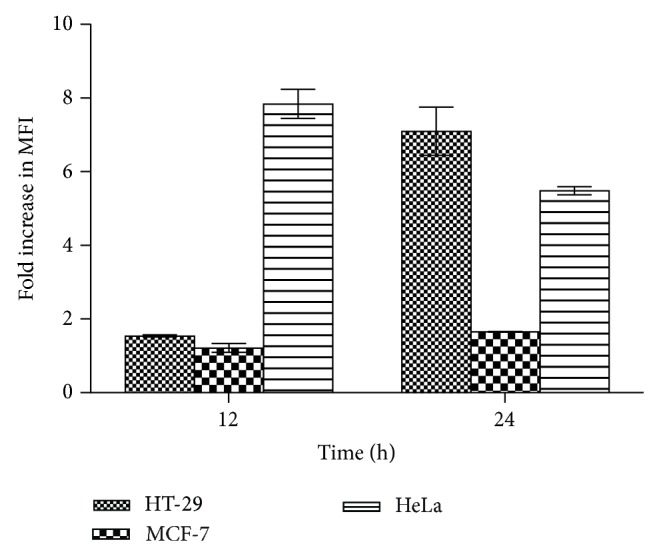
The graph shows fold increase in mean fluorescence intensity (MFI) of cells undergoing induction of ROS due to the treatment with the methanolic extract at 12 and 24 h. The results are means ± SD of three separate experiments evaluated using flow cytometry.

The effects on the phases of the cell cycle after 12 and 24 h exposure periods of cancer cells to 200 *μ*g/mL of extract were evaluated using flow cytometry. Representative cell cycle distribution histograms of HeLa cell are presented in Figure 8 whereas Figure 9 shows the percentage of cells in different cell cycle phases for HeLa, MCF-7, and HT-29 at 12 and 24 h. The results show that the extract induced significant accumulation of cells in G_0_/G_1_ phases and reduced the number of proliferating cells as shown by reduced S-phase at both 12 and 24 h for all the cells tested. Next, the ability of the extract (200 *μ*g/mL) at 12 and 24 h to induce reactive oxygen species (ROS) was evaluated using the cell permeant dye chloromethyl-2′,7′-dichlorofluorescin diacetate (CM-H_2_DCFDA). As shown in Figures 10 and 11, cells treated with* Holarrhena floribunda* extract (black panel) showed increases in ROS concentration compared to the untreated control (pink panel) in a time-dependent manner. The ROS induction effect of the extract was pronounced in HeLa cells at 12 h while in HT-29 at 24 h. The induction of ROS in MCF-7 is significantly low compared with other cancer cell lines.

## 4. Discussion

The use of plants as a source of human therapeutic medicine is as old as recorded history. The importance of plants as agents of therapeutic components is increasingly being recognized in line with current advances in technology. Globally, natural plant compounds have attracted attention as alternative therapeutic strategies in the fight against diseases, primarily because of their low toxicity and high therapeutic index [16, 17]. Many existing and contemporary drugs in clinical use are derived from the natural plants [7].* Holarrhena floribunda* leaves are an important source of drugs used in traditional medicine to cure different diseases, including diabetes, malaria, cancer, and oxidant damage related diseases [18, 19]. The present study evaluated the anticancer activity of the* Holarrhena floribunda* methanolic leaf extract in breast cancer cell (MCF-7), colon cancer (HT-29), cervical cancer (HeLa), and normal human fibroblast cell (KMST-6). The results of this study show that the extract exhibited cytotoxic effects towards all the cancer cell lines in a dose- and time-dependent manner. The IC_50 _values obtained for the various treatment protocols demonstrate that HeLa cells are more sensitive to the cytotoxic activity of the plant while KMST-6, a normal human fibroblast cell line, showed less sensitivity to the extract. The potential of the anticancer activity of the extract to discriminate between normal and cancer cells is an important paradigm in the design and discovery of chemotherapeutic agents. Consistent with this concept, trypan blue dye exclusion and colony formation assays confirm the antineoplastic activities of the extract against cancer cell lines compared to the normal KMST-6 human fibroblast cell line.* Holarrhena floribunda* is known to be rich in several phytochemicals like alkaloids, flavonoids, tannins, and cardiac glycosides. Some of these phytochemicals have been reported to possess antineoplastic activities against different cancer cell lines. Lamchouri et al. [20] and Hoet et al. [21] showed that the antiproliferative activities of* Peganum harmala* seeds and* Cassytha filiformis*, respectively, were due to their alkaloid constituents. Flavonoid activities against various cancers have also been reported [22–24].

To further elucidate the pathways of the cell death induced by the extract, phosphatidylserine flipping was evaluated using the APOPecerntage and Annexin/PI flow cytometric assays. Exposure of phosphatidylserine on the external surface of the cell membrane is generally accepted as one of the biomarkers of apoptosis [25]. The results demonstrated the concentration-dependent apoptotic-inducing potential of the extract. As a necessary corollary of the results of the cytotoxicity assay, HeLa cell showed a significant sensitivity to the extract compared to the other cell lines tested. The Annexin/PI assay also confirmed the ability of the extract to induce early and late apoptosis. Unlike necrosis, apoptosis is an important cell death mechanism that does not trigger an inflammatory response that occasions collateral destruction of normal cells in the surrounding microenvironment [26]. Thus, apoptosis is a protective mechanism that maintains tissue homeostasis by removing ailing cells [27]. Cancer cells, however, exhibit resistance to apoptosis in order to sustain their uncontrolled proliferation and, therefore, any apoptosis modulating compound is desirable as a plausible chemotherapeutic agent against cancer [28].

Two basic pathways involved in apoptosis are intrinsic (mitochondrial) and extrinsic (death receptor) pathways [29]. Caspase-3/7 is one of the effector caspases that is involved in the final execution of dying cells while caspase-9 is an initiator caspase that is involved in the intrinsic pathway [26, 27]. To understand the mechanism of action induced by the extract, caspase-3/7 and caspase-9 activities were evaluated. The results showed that the extract induced concentration-dependent increases in caspase-3 activity in HeLa cell lines while in contrast a concentration-dependent decrease in such activity was observed in MCF-7 and HT-29 cells. Caspase-9 results showed a similar trend of decrease in activities in all the cell lines. The possible reason for these observed results can be explained in two ways. The first is that the increase in caspase-3 activity observed in HeLa cells suggests that the extract induced apoptosis is a caspase-dependent manner while the decrease in caspase activities in MCF-7 and HT-29 cells presumably involves degradation of the protease, although the mechanisms of apoptosis induction need to be clarified.

However, in agreement with antiproliferative activity of the extract, the results of the cell cycle evaluation show that the extract arrests cell cycle progression by significantly restricting cells in G_0_/G_1_ phase. This implies that the extract perturbs the protein synthesis that is important to cell progression from G_1_ to S-phase. It is known that p53 and MDM2 proteins are important to the progression of the cell cycle at G_0_/G_1_ [30, 31]. It may be possible that the extract plays a role in the disturbance of these proteins, but this aspect was not investigated in this study. The effect of the extract on cell cycle progression may be due to its phytochemical constituents such as flavonoids and alkaloids.

Cells are known to thrive in low levels of reactive oxygen species (ROS), but a relative increase in ROS induces cell cycle arrest and apoptosis [24]. ROS-modulating drugs are, however, being proposed as therapeutic strategies to selectively target the destruction of cancer cells [32]. The results of this study indicate that the extract induced a time-dependent increase in ROS production. ROS production due to extract (200 *μ*g/mL) treatment for 12 and 24 h is more evident in HeLa cells which may explain why this cell line is more sensitive to the extract with regard to its antiproliferative, apoptotic, and cell cycle arrest effects.

## 5. Conclusion

Taken together, the results of this study clearly show that the extract was able to induce growth inhibition, apoptosis, cell cycle arrest, and induction of ROS in cancer cells. The compelling result shows that the extract contains possible anticancer bioactive compounds that require isolation and further characterization.

## Figures and Tables

**Table 1:** IC_50_ values of the methanolic leaf extract of *Holarrhena  floribunda* effects on HT-29, HeLa, MCF-7, and KMST-6 cell lines for 24, 48, and 72 h treatments. The IC_50_ value was obtained from the log dose cytotoxic effects of the extract using GraphPad Prism 6 statistical software.

Time (h)	HT-29 (*µ*g/mL)	HeLa (*µ*g/mL)	MCF-7 (*µ*g/mL)	KMST-6 (*µ*g/mL)
24	349.2	182.6	357.6	376.9
48	217.5	127.4	244.3	428.2
72	159.4	106.7	126.7	342.3

**Table 2:** The percentage of cell populations in different stages (live, apoptotic, and necrotic) following the extract treatment and evaluated by double staining in Annexin V-FITC/propidium iodide using flow cytometric assay.

	HeLa			MCF-7			HT-29		
	12 h	24 h	48 h	12 h	24 h	48 h	12 h	24 h	48 h
Live (%)	92.41 ± 0.03	84.94 ± 0.69	19.05 ± 0.51	95.22 ± 1.36	92.15 ± 1.77	7.1 ± 1.23	94.22 ± 2.23	78.45 ± 2.25	83.43 ± 4.61
Early apoptosis (%)	3.41 ± 0.32	6.26 ± 0.50	3.41 ± 0.36	4.69 ± 1.27	6.11 ± 0.34	0.15 ± 0.01	4.56 ± 1.60	16.72 ± 1.64	0.01 ± 0.01
Late apoptosis (%)	3.53 ± 0.12	6.53 ± 0.78	33.47 ± 1.90	0.18 ± 0.02	0.52 ± 0.19	3.93 ± 1.58	0.26 ± 0.04	4.46 ± 0.70	0.46 ± 0.16
Dead (%)	0.66 ± 0.23	1.38 ± 1.20	43.60 ± 1.75	0.03 ± 0.02	0.16 ± 0.03	87.60 ± 1.17	0.05 ± 0.01	0.07 ± 0.01	16.42 ± 1.73

## References

[1] Maxwell Parkin D., Bray F., Ferlay J., Pisani P. (2001). Estimating the world cancer burden: Globocan 2000. *International Journal of Cancer*.

[2] Siegel R., Naishadham D., Jemal A. (2013). Cancer statistics, 2013. *CA: Cancer Journal for Clinicians*.

[3] Yaacob N. S., Hamzah N., Nik Mohamed Kamal N. N. (2010). Anticancer activity of a sub-fraction of dichloromethane extract of *Strobilanthes crispus* on human breast and prostate cancer cells *in vitro*. *BMC Complementary and Alternative Medicine*.

[4] Leonard R. C. F., Williams S., Tulpule A., Levine A. M., Oliveros S. (2009). Improving the therapeutic index of anthracycline chemotherapy: focus on liposomal doxorubicin (Myocet). *Breast*.

[5] Wonders K. Y., Reigle B. S. (2009). Trastuzumab and doxorubicin-related cardiotoxicity and the cardioprotective role of exercise. *Integrative Cancer Therapies*.

[6] da Rocha A. B., Lopes R. M., Schwartsmann G. (2001). Natural products in anticancer therapy. *Current Opinion in Pharmacology*.

[7] Cragg G. M., Grothaus P. G., Newman D. J. (2009). Impact of natural products on developing new anti-cancer agents. *Chemical Reviews*.

[8] Wang H.-K., Morris-Natschke S. L., Lee K.-H. (1997). Recent advances in the discovery and development of topoisomerase inhibitors as antitumor agents. *Medicinal Research Reviews*.

[9] Lee K.-H. (1999). Novel antitumor agents from higher plants. *Medicinal Research Reviews*.

[10] Bouquet A., Debray M. (1974). Plantes medicinalis de Cote d' Ivoire. *Travaux et Documents de l'ORSTOM*.

[11] Kerharo J., Adam J. G. (1974). *Pharmacopee Senegalaise Traditionelle Plantes Medicinales et Toxiques*.

[12] Iwu M. M. (2014). *Handbook of African Medicinal Plants*.

[13] Abreu P. M., Martins E. S., Kayser O. (1999). Antimicrobial, antitumor and antileishmania screening of medicinal plants from Guinea-Bissau. *Phytomedicine*.

[14] Badmus J. A., Odunola O. A., Obuotor E. M., Oyedapo O. O. (2010). Phytochemicals and *in vitro* antioxidant potentials of defatted methanolic extract of Holarrhena floribunda leaves. *African Journal of Biotechnology*.

[15] Rieger A. M., Nelson K. L., Konowalchuk J. D., Barreda D. R. (2011). Modified annexin V/propidium iodide apoptosis assay for accurate assessment of cell death. *Journal of Visualized Experiments*.

[16] Sánchez-González P. D., López-Hernández F. J., López-Novoa J. M., Morales A. I. (2011). An integrative view of the pathophysiological events leading to cisplatin nephrotoxicity. *Critical Reviews in Toxicology*.

[17] Jung I. L. (2014). Soluble extract from *Moringa oleifera* leaves with a new anticancer activity. *PLoS ONE*.

[18] Badmus J. A., Odunola O. A., Yekeen T. A. (2013). Evaluation of antioxidant, antimutagenic, and lipid peroxidation inhibitory activities of selected fractions of *Holarrhena floribunda* (G. Don) leaves. *Acta Biochimica Polonica*.

[19] Fotie J., Bohle D. S., Leimanis M. L., Georges E., Rukunga G., Nkengfack A. E. (2006). Lupeol long-chain fatty acid esters with antimalarial activity from *Holarrhena floribunda*. *Journal of Natural Products*.

[20] Lamchouri F., Zemzami M., Jossang A., Settaf A., Israili Z. H., Lyoussi B. (2013). Cytotoxicity of alkaloids isolated from *Peganum harmala* seeds. *Pakistan Journal of Pharmaceutical Sciences*.

[21] Hoet S., Stévigny C., Block S. (2004). Alkaloids from *Cassytha filiformis* and related aporphines: antitrypanosomal activity, cytotoxicity, and interaction with DNA and topoisomerases. *Planta Medica*.

[22] Yadegarynia S., Pham A., Ng A. (2012). Profiling flavonoid cytotoxicity in human breast cancer cell lines: determination of structure-function relationships. *Natural Product Communications*.

[23] Matsuo M., Sasaki N., Saga K., Kaneko T. (2005). Cytotoxicity of flavonoids toward cultured normal human cells. *Biological and Pharmaceutical Bulletin*.

[24] Li H., Chen J., Xiong C., Wei H., Yin C., Ruan J. (2014). Apoptosis induction by the total flavonoids from *Arachniodes exilis* in HepG2 cells through reactive oxygen species-mediated mitochondrial dysfunction involving MAPK activation. *Evidence-Based Complementary and Alternative Medicine*.

[25] Fadok V. A., Voelker D. R., Campbell P. A., Cohen J. J., Bratton D. L., Henson P. M. (1992). Exposure of phosphatidylserine on the surface of apoptotic lymphocytes triggers specific recognition and removal by macrophages. *Journal of Immunology*.

[26] Elmore S. (2007). Apoptosis: a review of programmed cell death. *Toxicologic Pathology*.

[27] Fan T.-J., Han L.-H., Cong R.-S., Liang J. (2005). Caspase family proteases and apoptosis. *Acta Biochimica et Biophysica Sinica*.

[28] Tor Y. S., Yazan L. S., Foo J. B. (2014). Induction of apoptosis through oxidative stress-related pathways in MCF-7, human breast cancer cells, by ethyl acetate extract of *Dillenia suffruticosa*. *BMC Complementary and Alternative Medicine*.

[29] Hsu Y. L., Chia C. C., Chen P. J., Huang S. E., Huang S. C., Kuo P. L. (2009). Shallot and licorice constituent isoliquiritigenin arrests cell cycle progression and induces apoptosis through the induction of ATM/p53 and initiation of the mitochondrial system in human cervical carcinoma HeLa cells. *Molecular Nutrition and Food Research*.

[30] Plasencia C., Dayam R., Wang Q. (2005). Discovery and preclinical evaluation of a novel class of small-molecule compounds in hormone-dependent and -independent cancer cell lines. *Molecular Cancer Therapeutics*.

[31] Tang Y.-Q., Jaganath I. B., Sekaran S. D. (2010). Phyllanthus spp. induces selective growth inhibition of PC-3 and mewo human cancer cells through modulation of cell cycle and induction of apoptosis. *PLoS ONE*.

[32] Lampiasi N., Azzolina A., D'Alessandro N. (2009). Antitumor effects of dehydroxymethylepoxyquinomicin, a novel nuclear factor-*κ*B inhibitor, in human liver cancer cells are mediated through a reactive oxygen species-dependent mechanism. *Molecular Pharmacology*.

