# Corrigendum to “Antiproliferative and Apoptosis Induction Potential of the Methanolic Leaf Extract of *Holarrhena floribunda* (G. Don)”

**DOI:** 10.1155/2020/4319146

**Published:** 2020-11-23

**Authors:** J. A. Badmus, O. E. Ekpo, A. A. Hussein, M. Meyer, D. C. Hiss

**Affiliations:** ^1^Department of Medical Biosciences, University of the Western Cape, New Life Sciences Building, Robert Sobukwe Road, Private Bag Box X17, Bellville, Cape Town 7535, South Africa; ^2^Department of Chemistry, University of the Western Cape, Chemical Sciences Building, Robert Sobukwe Road, Private Bag Box X17, Bellville, Cape Town 7535, South Africa; ^3^Department of Biotechnology, University of the Western Cape, New Life Sciences Building, Robert Sobukwe Road, Private Bag Box X17, Bellville, Cape Town 7535, South Africa

In the article titled “Antiproliferative and Apoptosis Induction Potential of the Methanolic Leaf Extract of *Holarrhena floribunda* (G. Don)”, there was figure duplication [1]. As raised on PubPeer [2], Figure 4 KMST-6 control and KMST-6 200 *μ*g/mL are the same.

The authors could not find the original images and confirmed that the photomicrographs were directly copied into a Microsoft Word document as their record of the analysis. One or both of the photomicrographs was used in error and they should be disregarded. However, the raw data (Supplementary Materials) reflect significant differences between KMST-6 colony counts for the control and the 200 *μ*g/ml treatment, supporting the results in the article.

## Supplementary Materials

Supplementary Materials Clonogenic assay results, June 2014.
